# Recent advances in functional genome analysis

**DOI:** 10.12688/f1000research.15274.1

**Published:** 2018-12-21

**Authors:** Roderic Guigo, Michiel de Hoon

**Affiliations:** 1 Centre for Genomic Regulation, The Barcelona Institute of Science and Technology, Barcelona, Catalonia, Spain; 2 RIKEN Center for Integrative Medical Sciences, Yokohama, Japan

**Keywords:** functional genomics, massively paralel sequencing

## Abstract

At the beginning of this century, the Human Genome Project produced the first drafts of the human genome sequence. Following this, large-scale functional genomics studies were initiated to understand the molecular basis underlying the translation of the instructions encoded in the genome into the biological traits of organisms. Instrumental in the ensuing revolution in functional genomics were the rapid advances in massively parallel sequencing technologies as well as the development of a wide diversity of protocols that make use of these technologies to understand cellular behavior at the molecular level. Here, we review recent advances in functional genomic methods, discuss some of their current capabilities and limitations, and briefly sketch future directions within the field.

## Introduction

Progress in genomics during the last decade has been largely dependent on the development of methods for massively parallel sequencing of large numbers (up to a few hundred million per experiment in some cases) of short DNA sequences, where a single sequence read is about 50 to 150 base pairs. These methods—often referred to as next-generation sequencing (NGS)—are used for genome sequencing, but their high throughput also makes them very attractive for functional genomics assays, where the primary goal is to annotate genome regions with their biological function. This often requires quantitative estimates, which typically are derived from read counts. A myriad of experimental protocols and the accompanying bioinformatic pipelines have been designed that leverage the high-throughput capabilities of NGS to monitor a wide variety of molecular phenotypes reflecting very diverse genomic functions.

In this review, we summarize the protocols that are most frequently applied in functional genomics. Although oligonucleotide array-based methods for genotyping and gene expression are still widely used for large population screens because of the low cost and straightforward bioinformatics, genome tiling arrays, which were instrumental in the emergence of functional genomics ^1^, have largely been substituted by sequencing-based methods. Our review thus focuses on these. We also discuss recently developed methodologies for large-scale perturbation experiments that can probe the functionality of genomic loci in a massively parallel fashion. We discuss the limitations of short read length, efforts toward developing long read sequencing technologies, and novel approaches to analyze the behavior of single cells and cells in the context of their tissue of origin. We conclude by discussing the role of large-scale international consortia and future directions in functional genomics.

## Genomic protocols

Chromatin can be divided into the transcriptionally active open euchromatin and the transcriptionally silent heterochromatin or closed chromatin. The ATAC-seq (assay for transposase-accessible chromatin using sequencing) protocol uses transposases acting on accessible chromatin to create DNA fragments, which then are sequenced to identify open genomic regions in a particular cell type or condition ^2^. The number of cells from which the DNA is extracted is important, as too few cells may cause excessive digestion, leading to reads mapping to closed chromatin regions, whereas an excess of cells can cause insufficient digestion, creating long fragments that may be difficult to sequence ^3^. The methylation status of DNA can be assayed by using endonuclease digestion by methylation-dependent restriction enzymes, by using bisulfite treatment to convert unmethylated cytosine to uracil (which is recognized as thymine during sequencing), or by using methylation-dependent antibodies to immunoprecipitate methylated chromatin followed by sequencing (ChIP-seq) ^4^. False positives may occur if unmethylated cytosines fail to convert to uracil and are interpreted as methylated cytosines. It is essential to minimize DNA degradation during bisulfite treatment, as DNA fragmentation will hamper polymerase chain reaction (PCR) amplification. Tet-assisted bisulfite sequencing ^5^ can be used to resolve 5-methylcytosine and 5-hydroxymethylcytosine, both of which are found in mammalian genomes ^6, 7^ but are indistinguishable in traditional bisulfite sequencing. ChIP-seq-based approaches do not give single-nucleotide resolution of DNA methylation and are affected by genomic variations in CpG density.

Based on the availability of a suitable antibody, ChIP-seq can also be used to assay histone modifications ^8^ or the binding affinities of transcription factors to genomic regions ^9, 10^. Improvements to the ChIP-seq protocol have allowed greater resolution while reducing the number of cells required ^11, 12^. The use of a sensitive but specific antibody is essential for obtaining high-quality ChIP-seq data. Genomic assays typically produce an “epigenomic profile” along the genome (by mapping reads obtained by the assay to the genome). To combine and integrate epigenomic profiles produced by different assays (for instance, ChIP-seq from multiple histone modifications), segmentation algorithms are employed (for instance, 13, 14). These algorithms partition the genome into regions (segments) of similar profiles over all assays.

## Transcriptome protocols

Quantitative profiling of the transcriptional output of the genome relies on sequencing libraries of cDNA fragments derived from RNA (RNA-seq) ^15^. Sequence reads are subsequently assembled bioinformatically to reconstruct the full-length transcript sequence ^16, 17^ and to quantify the expression levels of annotated genes ^16, 17^. Cap analysis gene expression (CAGE), in contrast, specifically sequences the 5′ end of transcripts to pinpoint the transcription start site and therefore the promoter region of each transcript ^18^. CAGE uses a random oligonucleotide as the primer for the reverse transcription reaction, instead of an oligo-dT primer against the poly(A)-tail, allowing both poly(A)+ and poly(A)− transcripts—such as certain long non-coding RNA (lncRNAs)—to be profiled. Specific RNA fractions can be selected for inclusion in the sequencing libraries depending on their size, the chemical group at their 5′ end, the presence or absence of a poly(A) tail, or subcellular localization in the cytoplasm, nucleoplasm, or chromatin fraction ^19– 21^. These fractions may correspond to different classes of RNA or different stages in the pathway of RNA synthesis and post-processing. Protocols to assess the RNA-interacting partners include ribosome profiling ^22^ to identify mRNAs undergoing translation as well as various protocols based on high-throughput sequencing of RNA after cross-linking and immunoprecipitation (CLIP-seq and its variants) ^23^ to find transcripts bound to a specific protein. RNA modifications ^24^ such as methylation of adenosine nucleotides ^25^ can be identified by methylation-dependent immunoprecipitation of RNA followed by sequencing ^26, 27^.

Profiling of short non-coding RNAs—in particular, microRNAs (miRNAs)—typically relies on the ligation of oligonucleotide adapters to the 3′ and 5′ end of the short RNA followed by reverse transcription and PCR amplification making use of primer sequences in the adapters ^28^. Performing ligation of the 5′ adapter after reverse transcription allows ligation to the 5′ end of the cDNA, avoiding biases due to the presence of any modifications at the 5′ end of the short RNA molecule ^29^. To avoid biases introduced by oligonucleotide adapter ligation to the 3′ end, polyadenylation of the short RNA may be used instead. However, a disadvantage of this approach is that it does not allow the identification of the exact 3′ end of the short RNA, which is unfortunate, as addition and removal of nucleotides—in particular, adenosines—to the 3′ end of miRNAs are prevalent and may play a regulatory role in miRNA stabilization and degradation ^30– 32^.

## Regulation of expression in the 3D context of the genome

The 3D structure of the genome in the nucleus provides the physical architecture for expression regulation. Hi-C libraries ^33^, which are produced by chemical cross-linking, DNA fragmentation, proximity ligation, and high-throughput sequencing of ligated fragments, have shown that chromatin is organized in topologically associating domains, each consisting of chromosome regions that frequently interact with each other, and chromatin looping in which distal regulatory regions such as enhancers and promoters come in close physical proximity to each other. ChIA-PET (chromatin interaction analysis by paired-end tag sequencing) ^34^ combines proximity ligation of DNA fragments with chromatin immunoprecipitation of specific proteins to globally detect the chromatin interactions in which they participate.

As transcripts produced at regulatory sites ^35, 36^ may mediate distal interactions ^37^, identifying the chromatin binding sites of regulatory RNAs is of central importance. Chromatin isolation by RNA purification (ChIRP-seq) ^38^ uses tiling oligonucleotides to pull down a specific lncRNA together with the protein and DNA bound to it, followed by sequencing of the DNA component to determine the exact binding positions of the lncRNA to chromatin. For unbiased mapping of RNA–chromatin interactions, on a genome-wide scale, several protocols related to Hi-C have been developed that rely on proximity ligation of DNA to RNA using a bivalent oligonucleotide linker molecule: MARGI (mapping RNA–genome interactions) ^39^, global RNA interactions with DNA by deep sequencing (GRID-seq) ^40^, and chromatin-associated RNA sequencing (ChAR-seq) ^41^.

## High-throughput genomic perturbations

Although chromatin features and transcriptional activity at a genomic region can be indicative of a biological role, ultimately the function of a genomic region is defined by its contribution to the phenotype. Perturbation studies, in which activation or inhibition of specific genomic regions or their products is followed by phenotypic or molecular assays of the cellular response, can be used to elucidate the functional role of genomic loci.

Recent developments in perturbation technologies now allow genomic perturbation experiments to be highly multiplexed, with thousands of knockdowns or activations occurring in parallel in a population of cells. High-throughput perturbation experiments have become feasible in particular because of the development of the CRISPR/Cas9 (clustered regularly interspaced short palindromic repeats/Cas9) technology ^42^ to edit or otherwise modify the genome at specific sites. Whereas programming zinc finger nucleases ^43^ and transcription activator-like effector nucleases (TALENs) ^44^ to target specific genomic sites required extensive protein engineering that limited the scalability of these approaches, CRISPR/Cas9 recognizes and cleaves a particular DNA site using only a single guide RNA, which can easily be mass-produced. Nucleotide insertions and deletions introduced near the double-strand break by non-homologous end-joining can abrogate the expression or function of protein-coding genes but may not be sufficient to fully abolish the function of lncRNAs. Therefore, dual-CRISPR systems have been developed that use a pair of guide RNAs to generate double-strand breaks on both sides of a gene of interest, followed by non-homologous end-joining to repair the lesion, resulting in a full deletion of the gene ^45, 46^.

The applicability of this technology can be extended beyond genome editing by replacing the endonuclease Cas9 with catalytically inactive Cas9 (dead Cas9, or dCas9), which binds to specific genomic sites without inducing cleavage. In CRISPR interference (CRISPRi) ^47^, tight binding of CRISPR-dCas9 to DNA prevents binding of the transcriptional machinery, effectively shutting down transcript production at the targeted site. Improvements to the knockdown efficiency were achieved by fusing a Krüppel-associated box (KRAB) repressor domain to Cas9 ^48^, inducing histone modification changes associated with repression of expression. Similarly, dCas9 fused to a transactivating domain can be used to induce gene expression at specific sites ^48^. Epigenome editing can be performed by fusing dCas9 with epigenome writers or erasers (for example, a histone demethylase) ^49^.

Second-generation CRISPR editing tools allow the conversion of specific nucleotides at the genomic target site without inducing a double-strand break (for example, to create a novel stop codon) ^50^.

Large-scale CRISPR-based screening relies on designing guide RNAs in large quantities and introducing them, usually using a lentiviral vector, into a population of cells at a sufficiently low multiplicity of infection, such that most cells will receive either zero or one guide RNA; puromycin selection is applied to exclude cells that were not infected. Typically, up to 10 guide RNAs are used for a single locus, allowing checks for reproducibility and off-target effects due to inadvertent binding of guide RNAs elsewhere in the genome. During cell culture, the proportion of cells containing guide RNAs that inhibit the expression of genes essential for cell growth will decrease. Therefore, genes involved in cell growth can be identified by quantifying, using NGS, the relative enrichment of each guide RNA after culturing ^51^. CRISPRi-mediated knockdowns followed by single-cell genome-wide expression profiling (Perturb-seq) ^52, 53^ can be employed to obtain detailed information on the global transcriptome response to the perturbation and to observe cell state changes that are not easily assessed in a straightforward cellular assay.

For functional genomics, perturbation technologies that target DNA have one inherent drawback: it is unclear whether any observed phenotype is due to the inhibition of regulatory DNA elements at the targeted locus or to any RNA product originating there. This ambiguity can be avoided by targeting specific transcripts directly using locked nucleotide antisense oligonucleotides (ASO gapmers) or by using small interfering RNAs (siRNAs) or small hairpin RNAs (shRNAs). Target RNAs bound by an ASO gapmer are recognized and cleaved by endonuclease RNase H, followed by degradation of the RNA. In contrast, the RNA interference (RNAi) pathway relies on processing of siRNAs and shRNAs by Dicer and uptake into the RNA-induced silencing (RISC) complex, which binds to the target RNA, causing its cleavage by Argonaute and degradation by cellular exonucleases. The relative performance of RNAi or RNase H-mediated knockdown depends in part on the cellular localization of the targeted transcripts in either the nucleus or the cytoplasm ^54^.

## Long read sequencing

Reconstruction of the full-length sequence of long DNA or RNA molecules from NGS sequence reads is challenging because of their short read length. Two main technologies are being developed for long read sequencing (that is, sequencing of single molecules several thousand base pairs long). The first relies on the ability to detect the incorporation of a single nucleotide in an elongating DNA chain, which is synthesized by a DNA polymerase attached to the template DNA molecule to be sequenced ^55^. The second makes use of the ability to detect changes in an electrical field that are induced when a single nucleotide in the DNA molecule passes through a pore structure ^56^. Long read technologies have been used for “de novo” genome sequencing ^57^, resequencing of the human genome ^58^, and phased diploid genome assembly ^59^. They have also been used for transcript characterization ^60^, since short reads are unable to fully resolve the exonic structure of alternative transcript isoforms, which usually share a substantial fraction of the sequence. One advantage of long read technologies is that they are able to directly sequence RNA molecules ^26, 61^, eliminating the biases induced during the generation of the cDNA libraries inherent to short read NGS.

## Capture methods

Because of the large dynamic range of genome activity (for instance, gene expression), unbiased sequencing of samples to interrogate function genome-wide may be ineffective in uncovering sites within the genome with weak activity. Therefore, depending on the purpose, NGS analysis may be restricted to targeted genomic loci captured using oligonucleotide probes. Capture has been performed, for instance, to identify and characterize the exonic structure of lowly abundant transcripts (lncRNAs, in particular) using both short ^62^ and long ^63^ read sequencing.

## Spatial transcriptomics

Protocols to prepare libraries for NGS typically require a fair amount of input material (RNA or chromatin), necessitating large numbers of cells to produce a single library. If the samples are obtained from heterogeneous biological sources, such as tissues and organs, they are likely to contain multiple distinct cell types, which interact in a highly structured anatomical environment. Bulk sequencing of these samples reflects an average across the populations of cells used and is unable to resolve the underlying cell types and their position within the tissue sample. Recently, spatially resolved omics methods have been developed with the aim of performing functional genomics assays while retaining positional information ^64, 65^. Traced back to single-molecule fluorescence *in situ* hybridization (FISH) ^66^, these methods have dramatically increased throughput by using massively parallel sequencing. For instance, histological sections have been positioned on arrayed reverse transcription primers with unique positional barcodes to maintain the two-dimensional positional information in subsequent RNA-seq analysis ^67^.

## Single-cell technologies

Population averages, such as obtained from bulk tissue sequencing, may further obscure the biological reality of cellular heterogeneity even among cells of the same type. For example, genes that appear to be lowly expressed on the basis of their population average may be highly expressed in a few cells in the population and exert their biological role only there. To capture the heterogeneity among cells in a population, newer protocols have been developed that dramatically lower the amount of input material required, allowing profiling of individual cells ^68^. Genome sequencing of single cells has been used to identify genomic changes in tumor samples and to track their evolution ^69– 74^.

Single-cell RNA profiling can assay 50 to 10 ^4^ cells in parallel per experiment, depending on the protocol used; typical coverages are 1,000 to 6,000 genes per cell in case of primary cells and 5,000 to 10,000 genes for cell lines ^75^. In plate-based methods such as STRT (single-cell tagged reverse transcription) ^76^, Smart-seq ^77^, and Smart-seq2 ^78^, 50 to 500 single cells are placed in individual wells in 96-well plates, and cell lysis and reverse transcription occur in each well. Alternatively, a microfluidic chip can be used for cell placement and reactions ^79^.

Cell selection is biased by the cellular size and condition, which affects the probability of a cell to be placed successfully in a well. In pooled approaches such as CEL-seq ^80, 81^, a unique barcode is applied to individual cells in the initial stages in the protocol. As the barcode is incorporated in the cDNA during reverse transcription, the cDNAs originating from different cells are distinguishable and can be pooled for PCR amplification. To further increase throughput and reproducibility, in massively parallel single-cell RNA-seq, cells are sorted by fluorescence-activated cell sorting (FACS) into more homogeneous subpopulations in a 384-well plate before single-cell sequencing ^82^. As a poly-T primer is typically used for reverse transcription to selectively include poly(A)-tailed transcripts into the library while avoiding ribosomal RNA, other untailed transcripts such as some lncRNAs may be missed. Alternative strategies to reduce the ribosomal RNA component include duplex-specific nuclease treatment ^83^ and using a template-switching reaction to enrich for 5′-capped transcripts ^84^. Droplet-based isolation of single cells ^85, 86^ enabled massive parallelization with numbers of cells in the range of 10,000 or more, albeit at lower sensitivity.

As an alternative to single-cell analysis, deconvolution methods can be employed to estimate the proportions of constituent cell types in bulk tissue transcriptomic samples on the basis of the expression of known cell type marker genes ^82, 87^.

## Functional genomics across species

Comparative genomics methods have also been widely used to identify and catalogue functional regions in genomes. The underlying assumption is that functional regions are more conserved through evolution than genomic regions devoid of function. Genome sequence comparisons are widely used, in particular, to annotate protein-coding genes in newly sequenced genomes. However, while protein coding genes are under strong selective pressure, other functional regions, such as lncRNA genes and regulatory regions, have levels of conservation that are often barely distinguishable from background conservation ^88^ and therefore genome comparisons are, in general, less helpful to annotate them. For instance, whereas 80% of human protein-coding genes can be detected in mouse, fewer than 10% of lncRNAs are conserved between the two species ^89^. Comparative transcriptomic studies help to further understand to what extent the patterns of gene expression and the regulatory networks are conserved across species ^90– 92^. Particular attention has been paid to comparisons between human and mouse given the unique role of the mouse as a model of human biology ^93, 94^. While gene expression levels averaged over RNA samples are correlated between human and mouse ^89^, substantial differences are found when comparing the transcriptome of homologous cell types ^95– 97^. Overall, conservation of the patterns of expression between human and mouse seems to largely depend on the gene. The expression of some genes varies considerably across species and little across organs and that of other genes varies more across organs than across species. The mouse is a particularly good model for the biology of these genes in humans ^98^.

## Outlook

Massively parallel sequencing has been at the core of most large-scale functional genomic projects to date ^36, 99– 103^. These projects have not only generated data resources that are widely used by the scientific community but also defined “de facto” community standards for experimental protocols and bioinformatic pipelines, placing genomics in general and functional genomics in particular within the reach of individual laboratories. As a result of advances in experimental and computational methods, functional genomics assays are rapidly expanding beyond cell lines and tissue samples, which were mostly profiled in these projects, to complex organs, conditions, primary cells, and even single cells. Indeed, the Human Cell Atlas consortium has formed to characterize each individual cell type in the human body ^104^. Functional genomics assays are also being increasingly performed across multiple individuals within a species, allowing investigators to specifically link genetic variants to molecular phenotypes, as well as across different species. Most of these analyses to date have focused on the identification of single-nucleotide polymorphisms (SNPs) that affect gene expression (expression quantitative trait loci) ^103, 105^ but other molecular phenotypes, such as methylation and histone modifications, have also been monitored ^106^. Moving toward personalized genomics, the EN-TEx pilot project is currently carrying out multiple functional genomics assays in multiple tissues extracted from a few donors ^107^.

How these molecular phenotypes propagate in turn through intermediate levels of organizational complexity (cells, tissue, organs, and so on) determines how genetic variation ultimately impacts the phenotypic traits of organisms. Thanks to advances in digital imaging, data on intermediate phenotypes, such as tissues and organs, have recently been made available with associated genomics and molecular data in areas as diverse as cancer ^108^ and neuropathology ^109^. The GTEx project ^103^ has produced high-resolution histological images for 20,000 human tissue samples, together with matching genome and transcriptome sequencing data. Methods to identify SNPs associated with image-derived organ phenotypes such as neuroanatomical traits (that is, cortical thickness ^110^) have been developed, and an upcoming challenge is to relate molecular phenotypes, as monitored by functional genomics, to organ phenotypes, as monitored by imaging technologies.

We believe that we are still in the infancy of genomic technologies and there is ample opportunity for progress. The ultimate goal is the real-time *in vivo* multi-omics characterization of all of the cells within a multicellular organism—without interfering with the system. In the path toward this ideal, less-invasive protocols and protocols to probe the multiple facets of genome function at single-cell resolution (that is, protocols to perform multiple functional genomics assays in the same cell) will certainly be developed, as will methods able to simultaneously integrate data monitoring the behavior of millions of cells. The computational challenges associated with the exponential growth of data, driven by genomic technologies, will be enormous. New algorithms as well as new developments in hardware that overcome the limitations of current technological solutions may need to be developed.
